# AU-Rich Element RNA Binding Proteins: At the Crossroads of Post-Transcriptional Regulation and Genome Integrity

**DOI:** 10.3390/ijms23010096

**Published:** 2021-12-22

**Authors:** Ahmed Sidali, Varsha Teotia, Nadeen Shaikh Solaiman, Nahida Bashir, Radhakrishnan Kanagaraj, John J. Murphy, Kalpana Surendranath

**Affiliations:** 1 Genome Engineering Laboratory, School of Life Sciences, University of Westminster, 115 New Cavendish Street, London W1W 6UW, UK; w1523183@my.westminster.ac.uk (A.S.); w1657795@my.westminster.ac.uk (V.T.); n.shaikhsolaiman@westminster.ac.uk (N.S.S.); w1625969@my.westminster.ac.uk (N.B.); k.radhakrishnan@westminster.ac.uk (R.K.); 2 School of Life Sciences, University of Bedfordshire, Park Square, Luton LU1 3JU, UK

**Keywords:** Adenine-Uridine rich element, RNA binding proteins, replication stress, genome stability, DNA damage response, post-transcriptional regulation, oncogenes, tumour suppressors, cancer

## Abstract

Genome integrity must be tightly preserved to ensure cellular survival and to deter the genesis of disease. Endogenous and exogenous stressors that impose threats to genomic stability through DNA damage are counteracted by a tightly regulated DNA damage response (DDR). RNA binding proteins (RBPs) are emerging as regulators and mediators of diverse biological processes. Specifically, RBPs that bind to adenine uridine (AU)-rich elements (AREs) in the 3′ untranslated region (UTR) of mRNAs (AU-RBPs) have emerged as key players in regulating the DDR and preserving genome integrity. Here we review eight established AU-RBPs (AUF1, HuR, KHSRP, TIA-1, TIAR, ZFP36, ZFP36L1, ZFP36L2) and their ability to maintain genome integrity through various interactions. We have reviewed canonical roles of AU-RBPs in regulating the fate of mRNA transcripts encoding DDR genes at multiple post-transcriptional levels. We have also attempted to shed light on non-canonical roles of AU-RBPs exploring their post-translational modifications (PTMs) and sub-cellular localization in response to genotoxic stresses by various factors involved in DDR and genome maintenance. Dysfunctional AU-RBPs have been increasingly found to be associated with many human cancers. Further understanding of the roles of AU-RBP_S_ in maintaining genomic integrity may uncover novel therapeutic strategies for cancer.

## 1. Introduction

**Figure 1:**
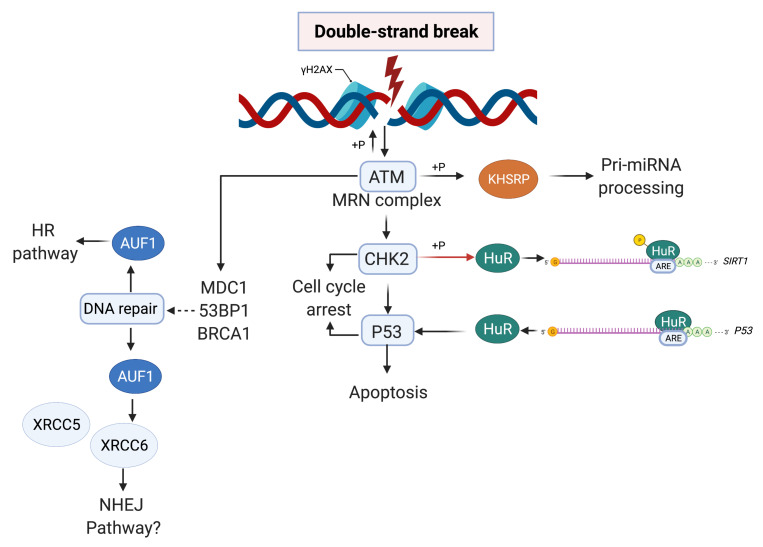
AU-RBPs at the nexus of the double strand break repair. DSBs initiated by endogenous or exogenous sources (red bolt) primarily lead to the activation of ATM. Initially, exposed DNA ends are sensed by the Mre11-Rad50-Nbs1 (MRN) complex, a key regulator of ATM activation, resulting in phosphorylation (+P) and activation of ATM. Activated ATM, phosphorylates downstream effectors CHK2, p53, BRCA1, and 53BP1 mediating cell-cycle arrest, apoptosis, and DNA repair through homologous recombination (HR) or non-homologous end-joining (NHEJ) pathways. ATM-directed phosphorylation of histone 2AX at serine 139 (γH2AX) is recognised by MDC1, resulting in wide-spread activation of γH2AX over chromatin domains recruiting DNA repair factors at the site of DNA damage. AU-RBPs have been reported to form direct interactions, such as, KHSRPs phosphorylation by ATM early in the DSB pathway promoting KHSRP’s role in pri-miRNA processing. AU-RBP HuR is phosphorylated by CHK2 promoting destabilsation of target mRNA *sirt1*. Alternatively, HuR may also stabilise p53 mRNA, promoting p53 translation to coordinate the DDR. AU-RBP AUF1 has been shown to associate with DSB repair through HR and may also interact with proteins involved in NHEJ, such as XRCC5 and XRCC6 (discussed below).

**Figure 2:**
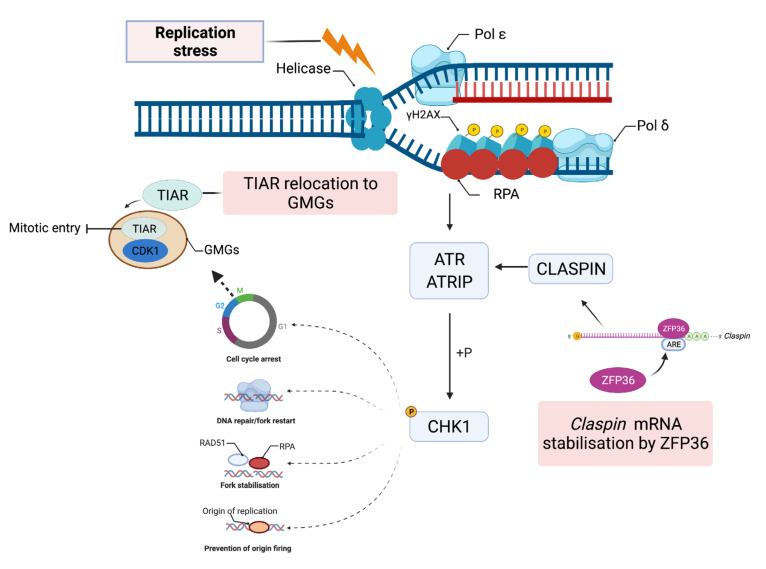
AU-RBPs at the nexus of the replication stress response. The eukaryotic replicative helicase complex unwinds parental DNA enabling replication of the leading and lagging strands by DNA polymerase Pol ε and Pol δ respectively. Replication stress can result in stalled replication fork progression (orange bolt) leading to stretches of single-stranded DNA (ssDNA) as the DNA continues to unwind. ssDNA becomes bound and protected by the ssDNA binding protein replication protein A (RPA), initiating ATR kinase recruitment. ATR recruitment is facilitated by interactions with ATR interacting protein (ATRIP), resulting in downstream activation of factors involved in the resolution of compromised replication forks. For ATR to phosphorylate its primary target, the master regulator CHK1 and CLASPIN must bind to RPA enabling phosphorylation of CHK1 at serine residues 317 and 345. This is accompanied by the phosphorylation of H2A histone family member x (H2AX) at serine 139 (γH2AX) early in the replication stress response. Activation of CHK1 ensures faithful restoration of the replication fork by initiating cell-cycle arrest, preventing origin firing, promoting fork stabilisation, DNA repair, and fork restart. The AU-RBP ZFP36 has been reported to be a key component in mediating faithful activation of CHK1 by ATR, by increasing the stability of CLASPIN mRNA. AU-RBP TIAR has been linked to arresting cell cycle at the G2/M border in response to replication stress by attenuating CDK1 in G2/M transition granules (GMGs) inhibiting entry into mitosis (discussed below).

Living cells are constantly engaged in a battle to alleviate and prevent DNA lesions that compromise genome integrity. It is estimated that each nucleated cell in the human body experiences more than 100,000 lesions per day [1]. Approximately 75% of these lesions are due to endogenous sources and occur following oxidative damage, base hydrolysis or as intermediates in DNA repair [1,2]. Furthermore, more deleterious lesions such as DNA double-strand breaks (DSBs) that occur less frequently arise as a consequence of exposure to both endogenous and exogenous sources of DNA damage. In particular, some DSBs can form when two single-strand DNA breaks (SSBs) are in close proximity or, following an encounter between the DNA replication machinery components with DNA lesions [3]. Many of these DNA lesions can be attributed to products of cellular metabolism, such as reactive metabolites, free radicals, and exposure to exogenous insults such as ultraviolet light (UV), ionising radiation (IR) and DNA damaging agents (as reviewed in [3]). Unrepaired lesions threaten the integrity of the genome and have been shown to serve as enablers of disease pathogenesis including cancers, neurodegenerative disorders and immunodeficiencies [4,5,6,7]. To maintain essential cellular functions and faithful transmission of genetic material to the next generation, cells harbour DNA damage response (DDR) mechanisms that continuously monitor and signal the presence of DNA damage at cell cycle checkpoints, activating pathways to initiate repair (Figure 1 and Figure 2). The key sensors of the DDR consist of proteins that identify DNA lesions resulting in the activation of DDR kinases upstream of a signaling cascade. Essential to the signaling cascade are mediator proteins that facilitate the phosphorylation of key factors that regulate transcription and the DDR network [8]. Effectors of the DDR are downstream substrates involved in a plethora of cellular activities, such as DNA replication, repair, and cell-cycle regulation; all of these activities are crucial for the maintenance of genome integrity [8].

## 2. The Eukaryotic DNA Damage Network

At the heart of DDR are three serine/threonine kinases of the phosphatidylinositol-3-kinase-like kinase family (PIKKs): ATM (ataxia-telangiectasia mutated), ATR (ATM and Rad3-related), and DNA-PK (DNA-dependent protein kinase) [9]. ATR and ATM are the master transducers of signaling through the DNA damage pathway and these kinases mediate the phosphorylation of their primary downstream effectors; checkpoint kinase 1 (CHK1) and checkpoint kinase 2 (CHK2) [10]. The number of DNA-PK targets is considerably less than those of ATM or ATR, with DNA-PK primarily involved in DSB repair [9]. Multiple studies have demonstrated that the distinct characteristics of ATR and ATM are partially attributed to the nature of DNA damage [11,12,13]. ATM is activated in response to DNA double-strand breaks (DSBs) that are detected by meiotic recombination 11 homolog 1 (MRE11), ATP-binding cassette–ATPase (RAD50), and phosphopeptide-binding Nijmegen breakage syndrome protein 1 (NBS1) forming the MRE11-RAD50-NBS1 (MRN) complex. Formation of this complex leads to the recruitment and activation of downstream effectors associated with the ATM pathway (Figure 1) [11,14]. ATR becomes active in response to the formation of single stranded DNA (ssDNA) commonly induced by DNA replication stress, resected DSBs and junctions between ssDNA and double-stranded DNA that are coated in heterotrimeric replication protein A (RPA) (Figure 2) [9,15]. Once activated, ATM and ATR result in the downstream activation of CHK1 and CHK2 that inhibit cyclin-dependent kinase (CDK) activity through multiple mechanisms [16,17]. CDK inhibition is required to slow down or inhibit cell cycle progression at specific cell cycle checkpoints: G1-S, intra-S, and G2-M [18]. Furthermore, signaling by ATM and ATR in the event of DNA damage upregulates the expression of DNA repair genes and mediates the recruitment of DDR proteins to sites of DNA damage through specific post-translational modifications such as phosphorylation, sumoylation, or acetylation [19]. The functional significance of many ATR/ATM-mediated phosphorylation events associated with DDR remains to be characterized [18,20]. Additionally, the expression of genes associated with DDR must also be tightly regulated to mediate an effective DDR as well as DNA repair. Extensive studies of the molecular networks of the DDR have also led to insights into dysregulation of these processes associated with cancer (as reviewed in [21]).

## 3. Multifunctional AU-RBPs Maintain Genome Integrity

In response to DNA damage, DDR orchestrates several processes, such as transcription inhibition, repression of 3′-end processing of mRNAs [22,23], reduction in the stability of mRNAs [24] and inhibition of translation factors [25] that are associated with initiation and elongation [26]. DNA damage is therefore associated with repression of gene expression that can be partially attributed to reduction in messenger RNA (mRNA) levels [27]. Additionally, global reduction in cell protein levels are also observed in the event of DNA damage, favouring translation of a subset of genes required during DDR [28]. For example, Microarray analysis performed on polysome-bound mRNAs demonstrated that, in response to DNA damage, DDR protein encoding mRNAs, selectively evade translational repression [28,29,30]. Pivotal to maintaining the expression of DDR genes upon DNA damage are RNA binding proteins (RBPs). RBPs modulate gene expression through the formation of ribonucleoprotein (RNP) complexes. This is achieved by their interactions with RNAs through sequence or structure specificities that are commonly located within mRNA untranslated regions (UTRs) and open reading frames (ORF) [31,32], as well as in pre-mRNAs in the nucleus.

This review focuses on presenting emerging evidence of multi-faceted roles for eight selected adenine-uridine (AU)rich element (ARE) RNA binding proteins (AU-RBPs) (Table 1) in maintaining genome integrity. Up to 8% of mRNA transcripts contain cis-acting ARE regulatory elements located within the 3′UTR [33]. AU-RBPs are historically categorised as functioning in mRNA post-transcriptional regulation by virtue of their ability to bind to AU-rich regions in the 3′UTR of mRNAs and mediate either mRNA degradation or stabilisation (reviewed in [34]). They contain a diverse repertoire of biochemically and structurally characterised RNA-binding domains (RBD) that vary in structural arrangement. AU-RBPs utilise their RBD(s) to achieve ARE binding utilising canonical RBDs, such as the RNA recognition motifs (RRM), hnRNP K-homology (KH type I and type II) domains and CCCH zinc finger motifs [35]. Certain RBPs are not only limited to interacting with mRNA but have also been reported to interact with DNA. For example, KH type I and type II RBDs of AU-RBP KHSRP also exhibit binding to ssDNA (reviewed in [36]) and the RRM of AUF1 has been reported to also interact with the single-stranded human DNA telomeric repeat sequence d(TTAGGG)n [37]. Furthermore, AU-RBPs may contain one or several RBDs indicating their versatility to interact with multiple targets associated with various biological events [38,39,40]. AU-RBPs therefore maintain key roles in RNA metabolism regulating RNA maturation, stabilisation, degradation, surveillance and translation [41].

Dysfunctional AU-RBPs are linked to multiple diseases such as, neurodegenerative disorders, cardiovascular disease and cancer [42,43,44]. In addition to presenting evidence for the involvement of AU-RBPs in human diseases, we also explore reported involvement of these proteins in the maintenance of genome integrity [45,46,47,48].

## 4. Post-Transcriptional Regulation of DDR Genes by AU-RBPs

Numerous studies have reported that, in the event of DNA damage, AU-RBPs are able to modulate the expression of selected DDR genes. For example, Hu antigen R (HuR) binds to, stabilises and subsequently increases translation of a plethora of mRNA targets essential for cell cycle control and DNA repair such as; Cyclins (D1, E1, A2, B1), p53, and BRCA1 [60,90]. Furthermore, alterations to HuR activity in response to DNA damage have also been reported to modulate the expression of genes involved in the DDR [91]. HuR was reported to bind and stabilise *p53* mRNA in a manner dependent on exposure to short-wavelength UV light (UVC) in human colorectal carcinoma cells (Figure 1) [61]. Overexpression of HuR was shown to increase the abundance of p53, whereas HuR depletion diminished p53 translation [61]. Recently, HuR has been reported to reduce radiation-induced DNA damage through post-transcriptional regulation of NHEJ factor AT-rich interactive domain 1A (SWI-like) (ARID1A) a factor that promotes NHEJ, by increasing expression of ARID1A in response to ionising radiation [51,92]. Other AU-RBPs have also been shown to be important regulators of p53. In response to DNA damage-induced during B cell development, AU-RBP TIA1 dissociates and relocates p53 mRNA from stress granules to polysomes, allowing its rapid translation, required for generating a functional B cell-receptor [93]. Using TIA-in-vivo UV crosslinking and immunoprecipitation (TIA-iCLIP) we identified a subset of known p53-targeted genes that contains multiple binding sites and motifs available for TIA1 interaction in their 3′UTR region of mRNA transcripts [75]. Interestingly, TIA1 protein is found to be immunoprecipitated with various p53-targeted cell-cycle regulator mRNAs, including NUP98, GADD45B and CDKN1A, validating TIA1 function in modulating post-transcriptional levels of these DNA-Damage related mRNAs [94]. Thus, TIA1 dissociates and relocates multiple target mRNAs following DNA damage elicited by UV.

TIAR has been recently reported to act as a novel component of the replication stress response and to be an important regulator of the G2/M transition required for arresting cells at the G2/M border (Figure 2) [74]. TIAR relocalises in stress induced nuclear foci, known as G2/M transition granules (GMGs) at stalled replication forks that contain components of the transcription, splicing, DNA replication, and repair machinery [74]. Furthermore, TIAR attenuates CDK1 activity by retaining CDK1 within the GMGs during replication stress, a mechanism that requires TIAR’s RNA binding capabilities [74]. During B cell activation, TIA1 sequesters p53 mRNA within stress granules in the cytoplasm, conferring translational repression of p53 mRNA [93].

AU-RBPs AUF1 and TIAR were reported to interact with the 3′UTR of Gadd45α (a transcript that is upregulated in response to stress stimuli) mRNA in response to genotoxic doses of methyl methanesulfonate (MMS). In unperturbed Hela cells, AUF1 was found to bind *Gadd45*α mRNA, reducing its stability, whereas TIAR inhibited *Gadd45*α translation. This association effect was shown to diminish following treatments with MMS, resulting in the dissociation of AUF1 and TIAR from *Gadd45*α mRNA thereby increasing its half-life and translation [95].

Recently ZFP36, a member of the TTP family of proteins, was shown to modulate ATR-CHK1 activation following DNA damage through stabilisation of *Claspin* mRNA, a mediator of ATR-CHK1 interactions (Figure 2). ZFP36-dependent *Claspin* mRNA stabilisation facilitated ATR–CHK1-dependent replication fork stability to maintain chromosomal stability. Furthermore, depletion of ZFP36 compromised subsequent ATR-CHK1 activation and negatively impacted genome integrity [76]. Indirect evidence for the role of the ZFP36 family proteins, ZFP36L1 and ZFP36L2, in the DDR has also come from studies on developing lymphocytes in mice [81,96]. Concomitant deletion of *zfp36pl1* and *zfp36l2* in early developing T cells in the thymus increased levels of γH2AX, a marker of DNA double-stranded breaks, and also increased phosphorylation of substrates of ATM/ATR kinases [81,96]. CRISPR-Cas9 knockout of *ZFP36L1* in U2OS osteosarcoma cell line models increased markers of replication stress, including mitotic chromosome breaks, chromosome missegregation, incidence of micronuclei and 53BP1 G1-nuclear bodies [97]. Thus, it is possible that zfp36l1 and zfp36l2 post-transcriptionally coordinate the expression of proteins involved in the DDR [96]. In support of this proposal [81,96], in vitro studies on human cell lines [98] have reported that ZFP36L1 and ZFP36L2 [98] can act as negative regulators of cell cycle progression by targeting numerous cell cycle associated mRNAs that have significant overlap with DDR genes, such as several Cyclins and CDKs [96].

In addition to the presence of AREs in the 3′ UTR of mRNAs, recent studies using transcriptome analysis techniques and computational biology approaches have revealed the existence of intronic AREs sequences present on pre-mRNAs in the cell nucleus [52,99,100,101,102,103]. To date, understanding the roles of intronic AREs in classic functions of AU-RBPs stabilising or destabilising RNA transcripts or effects on mRNA translation are in their infancy. There is, however, good evidence that HuR can function in RNA stabilisation of pre-mRNAs by virtue of intronic ARE binding [99,101,103]. For example, HuR knockdown in HEK-293 cells resulted in reduced levels of mRNA for genes containing intronic AREs [103]. Along with AREs present in the 3′UTR of mRNAs, the discovery of intronic AREs has revealed that greater than 50% of human genes may contain AREs [103]. The relevance of roles of intronic AREs for AU-RBP functions in maintaining genomic integrity is currently unknown. However, AU-RBPs (Table 1) are all nucleo-cytoplasmic shuttling proteins; therefore, some potential nuclear roles for AU-RBPs in maintaining genomic integrity could involve intronic AREs.

## 5. DNA Damage Induces Post-Translational Modifications of AU-RBPs

**Figure 3:**
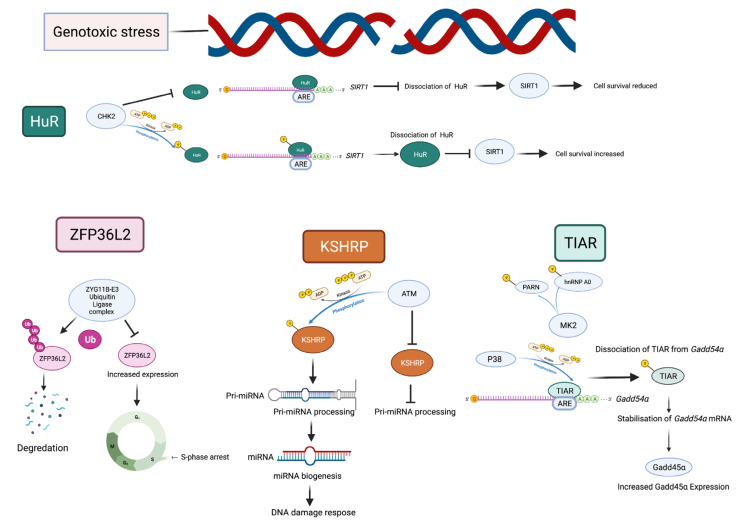
AU-RBPs exhibit post-translational modifications in response to genotoxic stress. Genotoxic stress results in post-translational modifications of AU-RPBs influencing their activity. The AU-RBP HuR is targeted for phosphorylation by CHK2 resulting in its dissociation from *SIRT1* inhibiting SIRT1 expression and increasing cell survival. When CHK2 mediated phosphorylation of HuR is blocked, HuR remains bound to *SIRT1* resulting in increased SIRT1 expression and decreasing cell survival. KSHRP is phosphorylated by ATM promoting its role in Pri-miRNA processing and miRNA biogenesis, which may have a role in the DDR. Concomitant phosphorylation of AU-RBP TIAR by p38 and PARN and hnRNP A0 by MK2 is involved in TIARs dissociation from *Gadd45α* resulting in *Gadd45α*’s stabilization and increasing GADD45α expression. Polyubiquitination of ZFP36L2 by ZYG11B-E3 ubiquitin ligase complex regulate ZFP36L2 expression levels ensuring its degradation. Genotoxic stress increases ZFP36L2 resulting in S-phase arrest which may be attributed to a reduction in polyubiquitination of ZFP36L2.

Post-translational modifications (PTMs) play an important role in mediating and sustaining DDR signalling [18]. Phosphoproteomic screens have demonstrated that DNA damage regulates the phosphorylation of several RBPs [20,104]. Furthermore, these phosphorylation events can be attributed to DDR proteins, such as DNA damage sensors (ATM, ATR, and DNA-PK), transducers (CHK1 and CHK2), and multiple downstream kinases. AU-RBPs listed (Table 1) are targeted for PTMs by DDR proteins in the event of DNA damage. CHK2 has been previously shown to regulate HuR’s interaction with target mRNA in response to genotoxic doses of hydrogen peroxide (H_2_O_2_) [105]. In human diploid fibroblasts, CHK2 phosphorylation of HuR was demonstrated to be an essential event in inducing its dissociation from the mRNA encoding the pro-cell survival factor *Sirtuin 1*(*SIRT1*), resulting in decreased SIRT1 expression (Figure 3) [105]. This process was shown to be largely mediated following phosphorylation of three HuR residues S88, S100 and T118 by CHK2 [105]. Specifically, S100 phosphorylation by CHK2 was shown to be essential for its dissociation from *SIRT1 mRNA*. In response to ionising radiation-induced DNA damage in B lymphocytes, HuR phosphorylation by CHK2 induced its association with mRNAs and thereby post-transcriptionally upregulated a number of proteins including ZFP36L1 [58]. HuR phosphorylation by CHK2 was further supported through microarray analysis in CHK2 proficient and deficient HCT116 colorectal carcinoma cells [57]. HuR was revealed to dissociate with target mRNAs in CHK2 proficient cells when exposed to ionising radiation, whereas HuR remained bound to mRNA targets in CHK2 −/− HCT116 cells [57]. Furthermore, mutations to HuR that inhibit its phosphorylation by CHK2 disrupted its dissociation from its mRNA targets following IR, reducing cell survival [57]. p38-mediated phosphorylation of the AU-RBP TIAR and concomitant MK2 (mitogen-activated protein kinase (MAPK)-activated protein kinase-2) mediated phosphorylation of hnRNP A0 and PARN was proposed to be involved in the regulation of Gadd45α levels in response to genotoxic stress [106]. TIAR’s dissociation from *Gadd45α* mRNA via p38 phosphorylation was demonstrated to be an important initiating factor in Gadd45α’s subsequent stabilisation and expression following genotoxic stress (Figure 3). Gadd45α blocks cell mitosis in the presence of unrepaired DNA. Specifically, RNA-IP of TIAR in doxorubicin-treated Hela cells demonstrated that TIAR’s binding to *Gadd45α* mRNA is directly correlated to p38 activity. In Hela cells with unperturbed p38, TIAR was able to dissociate from *Gadd45α*, whereas p38 inhibition restored TIAR’s binding to *Gadd45α*. Furthermore, in vitro kinase assays revealed strong phosphorylation of TIAR by p38 following genotoxic stress, implying that TIAR’s dissociation from *Gadd45α* is achieved following p38 mediated PTM of TIAR [106].

The AU-RBP, KH-type splicing regulatory protein (KHSRP) was reported to be targeted for phosphorylation by ATM in response to DNA double-strand breaks induced by the radiomimetic drug neocarzinostatin (NCS) [69]. Immunoprecipitated KHSRP in human ATM proficient and deficient fibroblast cells demonstrated KHSRP phosphorylation in ATM proficient cells, whereas no notable phosphorylation of KHSRP was detected in cells deficient for ATM. Furthermore, mutations to three putative ATM phosphorylation sites of KHSRP at Ser 132 (S132), Ser 274 (S274) and Ser 670 (S670) were revealed to impact KHSRP phosphorylation in ATM proficient cells. Specifically, mutations to S274 and S670 diminished ATM-mediated KHSRP phosphorylation greater than that of S132 mutations, implicating S274 and S670 as major ATM target residues [69]. Interestingly, ATM phosphorylation of KHSRP was shown to promote KHSRP activity, enhancing primary-microRNA (pri-miRNA) interactions in response to DNA damage and mutations to KHSRP’s ATM phosphorylation sites impaired its activity in miRNA biogenesis (Figure 3). Additionally, RNA-CHIP (chromatin immunoprecipitation) assays demonstrated that ATM phosphorylation is essential for mediating KHSRP interactions with pri-miRNAs following DNA damage, a process that was impaired following mutations to KHSRP’s ATM phosphorylation sites [69]. ATM-mediated phosphorylation of KHSRP is therefore essential for its role in pri-miRNA processing in response to DNA damage, which has been reported to be important in DNA repair (Figure 3) [107,108].

The AU-RBP ZFP36L2 was reported to be regulated in a cell cycle-dependent manner and accumulates in the mitotic phase of the cell cycle [86]. Downregulation of ZFP36L2 expression in the cell interphase was shown to be mediated post-translationally by polyubiquitination of the protein by ZYG11B-E3 ubiquitin ligase complex, resulting in its subsequent degradation. Furthermore, in cisplatin-treated cells, ZFP36L2 expression was increased and mediated cell S phase arrest protected cells from dying following cisplatin-induced DNA damage, implying that ZFP36L2 plays a role in controlling S phase transition under conditions of genomic instability [86].

## 6. Subcellular Localisation of AU-RBPs in Response to DNA Damage

**Figure 4:**
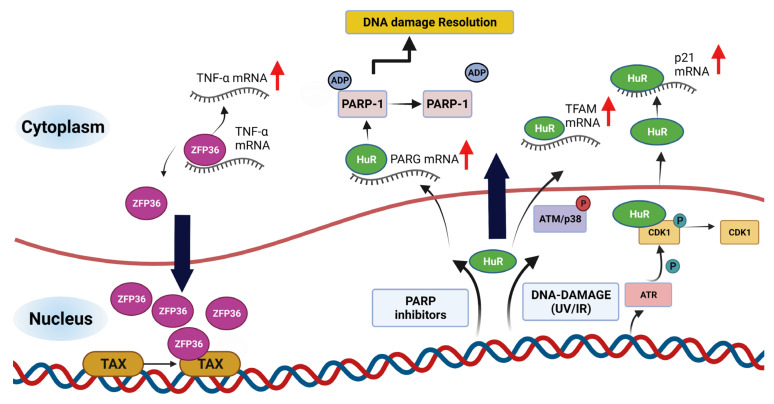
Subcellular environment dictates nucleocytoplasmic shuttling of AU-RBPs. In the nucleus, interaction of AU-RBP ZFP36 with retroviral transcriptional activator Tax protein results in shuttling of ZFP36 inside the nucleus and indirectly increase of TNFα mRNA in the cytoplasm. AU-RBP HuR translocates to cytoplasm in response to DNA-damage, resulting HuR binding and stabilisation of its mRNA targets including TFAM, PARG, CDKN1A. Bold black arrows indicate mobility of the AU-RBPs across the cellular compartments shown and red arrows indicate increased stability of the mRNAs.

Nucleocytoplasmic shuttling activity elicited by AU-RBPs may partially explain their multifaceted nature and emergence as key players in maintaining genome integrity [109]. Post-transcriptional modulation of gene expression largely occurs in the nucleus and cytosol (as reviewed in [110]). DNA damage can result in changes in AU-RBP localisation and such changes can determine the interactions and activities of AU-RBPs (Figure 4). Well characterised AU-RBPs, such as the ZFP36 family, contain both nuclear localisation sequences (NLS) and functional nuclear export sequences (NES), facilitating nuclear import and export, respectively [109,111]. Interestingly, it was reported that the AU-RBP ZFP36 acted as a transcriptional co-repressor of p65/NF-κB [112]. Furthermore, ZFP36 was shown to interact with the retroviral transcriptional activator Tax protein; this association with Tax induced nuclear accumulation of ZFP36 and indirectly increased TNFα expression (Figure 4) [113]. A recent report provided evidence that the accumulation of ZFP36L1 in the nucleus is cell cycle-dependent, peaks at G1/S and is dependent on the protein’s C-terminal serine-rich cluster [114].

The AU-RBP HuR has been shown to exhibit changes in subcellular localisation in a cell cycle-dependent manner. During the G1 phase, HuR was found to be primarily nuclear, whereas, in S and G2, HuR was located primarily in the cytoplasm in order to regulate the stability and translation of mRNA targets [63,115]. In response to UV damage, HuR subcellular localisation has been shown to be regulated in a CDK1 dependent manner. Specifically, cells exposed to UV activate ATR enabling nuclear-cytoplasmic shuttling of HuR [116]. ATR phosphorylation of CDK1 promotes its disassociation from HuR, enabling translocation to the cytoplasm where HuR binds to mRNA target p21/CDKN1A, reducing its turnover [116]. Recently, ATM/p38 signaling was shown to promote the cytoplasmic translocation of HuR from the nucleus in response to IR, stabilising mitochondrial transcription factor A (TFAM) mRNA [55].

HuR has also been reported to mediate efficient DNA repair through post-transcriptional regulation of Poly (ADP-ribose) glycohydrolase (PARG) mRNA [56]. In pancreatic ductal cancer cells treated with PARP inhibitors translocation of HuR from the nucleus to the cytoplasm is stimulated, promoting stabilisation of PARG mRNA. PARG coordinates efficient repair of DNA lesions together with Poly (ADP-ribose) polymerase 1 (PARP-1). Specifically, HuR stabilisation of PARG promotes the removal of Poly (ADP-ribose) (PAR) moieties from PARP-1 thereby initiating replication fork restart and DDR resolution (Figure 4) [117,118]. HuR silencing resulted in decreased mRNA half-life and expression of PARG, as well as increased susceptibility to double-strand breaks in the presence of PARP inhibitors veliparib and olaparib. Furthermore, diminishing PARG levels through HuR silencing was demonstrated to impact PARP-1 release from chromatin, suggesting deficiencies in the HuR/PARG axis increase PARP-1 accumulation on chromatin inhibiting the efficiency of DNA repair [56].

TIA-1 and TIAR also shuttle between the nucleus and cytoplasm and both play an important role in alternative splicing of pre-mRNAs in the nucleus [119]. TIAR was also reported to relocate from the nucleus to the cytoplasm in response to Fas-mediated apoptotic cell death [120]. It has also been reported that both TIA-1 and TIAR can bind to DNA with high affinity; they probably shuttle between RNA and DNA ligands in carrying out their cellular functions [121].

## 7. AU-RBPs at the Crossroads of DNA Repair and Genome Maintenance

Selected AU-RBPs (Table 1) have recently emerged as key mediators of DNA repair, eliciting canonical roles through mRNA regulation and non-canonical direct interactions at the sites of DNA damage where they are required for efficient DNA repair through regulating distinct repair pathways.

The AU-RBP AUF1 has been reported to localise at sites of DNA damage, mediating the efficient repair of DNA lesions, suggesting that these interactions can be achieved independently of mRNA interactions [48]. In proteomic screens for proteins involved in DNA-end resection, a process that produces ssDNA tails required for the invasion of a complementary DNA strand within the HR pathway [48,122], AUF1 was found to bind structures that mimic DNA end-resection intermediates composed of protruding nucleotide ends independent of RPA complexes [48]. Furthermore, AUF1 was reported to bind chromatin DNA purified from Hela cells, without AUF1’s RNA recognition motif involvement, suggesting that binding to DNA, as well as chromatin structures, is independent of AUF1 RNA regulatory mechanisms [48]. Intriguingly, CPT treatment in AUF1-depleted cells markedly reduced the efficiency of DNA end resection and RPA binding in comparison to control cells, while chromatin IP (ChIP) of FLAG-tagged AUF1 transfected into engineered Hela cells, expressing inducible AsiSI endonuclease to control DSBs in vivo, demonstrating the increased presence of AUF1 proximal to DSB sites [48,123]. Finally, AUF1 downregulation was demonstrated to reduce HR efficiency by approximately 50% in comparison to control cells, highlighting that AUF1 may indeed be involved in mediating efficient DNA repair through the HR pathway [48]. AUF1 has also been reported to bind factors involved in NHEJ. Liquid chromatography mass spectrometry analysis of immunoprecipitates, obtained from oral cancer cell lines SCC4 and MDA1986, demonstrated interactions with X-ray repair cross-complementing protein 5 and 6 (XRCC5 and 6 also known as Ku70 and Ku80) that bind broken DNA ends and mediate repair through NHEJ (Figure 1) [124,125].

**Figure 5:**
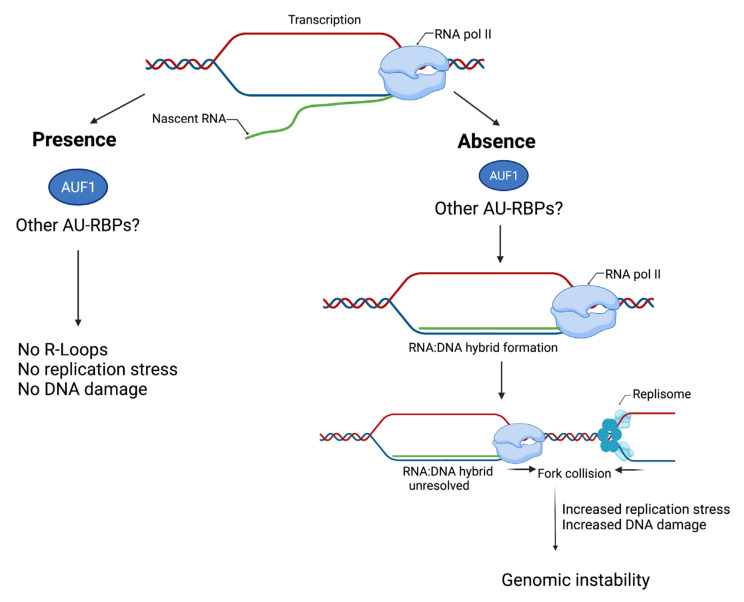
Potential roles of AU-RBPs in preventing R-loop formation in response to replication stress. During the process of transcription by RNA polymerase II (RNA pol II), the nascent RNA is prevented from hybridizing with the template strand DNA strand (RNA: DNA hybrid) in the presence of AUF1 and possibly other AU-RBPs. Therefore preventing the formation of R-loops that cause replication stress and DNA damage. In the absence of AUF1 and possibly other AU-RBPs the nascent RNA can hybridise with the template strand and displace the non-template strand into a long stretch of ssDNA forming an R-loop structure. Unresolved R-loops can cause transcription and replication fork collisions, resulting in increased replication stress and DNA damage that compromise genomic instability.

R-loops form through the hybridisation of nascent RNA with a template DNA strand, displacing the non-template strand into long stretches of ssDNA, forming non-canonical RNA–DNA hybrid structures (Figure 5) [126,127]. R loops play an important role in transcription termination, immunoglobulin class switching, mitochondrial DNA replication, chromatin modification, and telomere regulation dynamics [128]. However, unscheduled and unprocessed R-loop formation can prove deleterious to genome integrity, inducing hypermutagenesis due to the exposed ssDNA becoming susceptible to DNA modifying enzymes such as activation-induced cytidine deaminase (AID) and increasing the occurrence of DNA breaks as a result of replication stress or replication fork collision with the transcription machinery (transcription-replication conflicts) [129,130,131,132]. Importantly, the nascent RNA is fundamental to the formation of R-loops; dysfunction in factors involved in RNA biogenesis and processing induce the formation of R-loops [133,134,135]. Such factors may include selected AU-RBPs that may protect the nascent RNA through direct or indirect interactions, preventing its hybridization with the template DNA strand. A recent study utilising mass spectrometry analysis presented evidence for the AU-RBPs HuR and AUF1 being associated with the DNA: RNA hybrid-interactome [53]. Furthermore, in vitro studies revealed that depletion of AUF1 resulted in a defective DNA damage response and also increased the number of R-loops in human Hela cells following DNA damage (Figure 5) [48]. Overexpression of the Rnase H1 led to a reversal of the phenotype associated with AUF1 depletion, suggesting a role for the resolution of R-loops in rescuing a defective DDR [48]. These results indicate that AU-RBPs may play a role in deterring R-loop formation similar to what has been reported for other RBPs [46,136].

Evidence suggests that some genomic regions known as common fragile sites (CFS) are susceptible to gaps and breaks (termed ‘expression’) in response to replication stress and could be a source of genomic instability present in some cancers [137]. In vitro, CFSs are usually stable under unperturbed growth conditions and can become expressed following mild replication stress when treated with low doses of aphidicolin or in vivo by dysfunctional oncogene activation. Furthermore, CFS are prone to mutations and are susceptible to sister chromatid exchange exhibiting translocation and deletions [138]. Breakpoints in tumours have been mapped to CFS and result in the loss of tumour suppressor genes or amplification of oncogenes, which are hallmarks of cancers [139,140]. Unlike CFS present in all individuals, a separate classification of fragile sites known as rare fragile sites exists in less than 5% of the population [141]. Furthermore, it has also been determined that replication blocks at CFS loci, as a result of transcription replication conflicts, may lead to the formation of R-loops, leading to breaks, suggesting that the modulation of R-loops is essential to maintaining CFS stability [142]. Interestingly, four of the eight AU-RBP genes discussed in this current review (Table 1) are located within known fragile sites [143]. Specifically, the *ZFP36* gene is mapped to the common fragile site FRA19A, and the *ZFP36L1* gene is located on the common fragile site FRA14C. Furthermore, *TIAR* and *HuR* genes are mapped to rare fragile sites FRA10A and FRA19B, respectively. The occurrence of *ZFP36*, *ZFP36L1*, *TIAR*, and *HuR* at fragile sites is intriguing; however, the significance of this in relation to R loop formation and genome integrity is currently unknown. Intriguingly, interstitial deletions in del(14)(q) close to the *ZFP36l1* locus are a recurrent aberration in B cell malignancies [144].

## 8. AU-RBPs in Cancer

**Figure 6:**
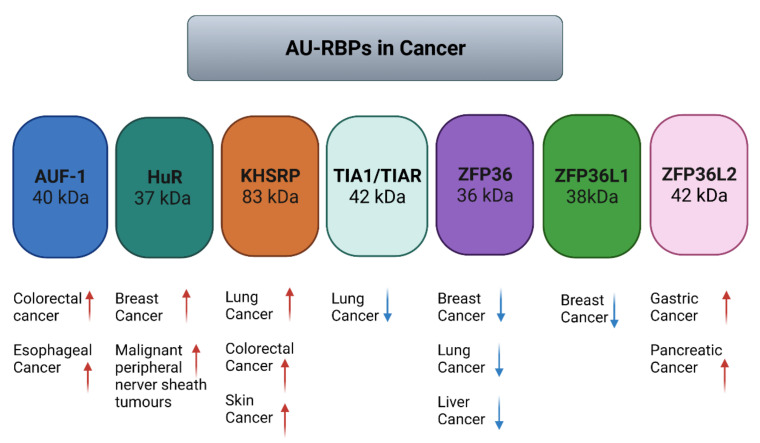
AU-RBPs are aberrantly expressed in various kinds of cancer, where AUF1, HuR, KHSRP and ZFP36L2 are found to be mostly upregulated and TIA1, ZFP36 and ZFP36L1 are found to be downregulated in breast, lung and liver cancers.

Multiple processes are employed by AU-RBPs to maintain genome integrity (Table 1); therefore, it is unsurprising that dysfunctions of AU-RPBs are associated with various diseases, including cancer. Numerous studies have provided a large body of evidence for a variety of roles of AU-RBPs in different cancers and the evidence presented here is selected information rather than an exhaustive survey (Figure 6) (Table 1). AUF1 is overexpressed in esophageal squamous cell carcinoma patient tissue samples [49] and AUF1 overexpression has also been shown to promote colorectal cancer progression and is an indicator of poor prognosis in this disease [50]. HuR is elevated in clinical ductal invasive carcinoma (DIC) and ductal carcinoma in situ (DCIS) breast cancer samples [64,65]. Elevated cytoplasmic expression of HuR was found to be a possible indicator of poor prognosis in breast cancer patients that received paclitaxel- and anthracycline-based neoadjuvant chemotherapy (NACT) [66]. Furthermore, HuR has been reported to be an oncogenic driver for malignant peripheral nerve sheath tumours (MPNSTs) [67]. KHSRP has been reported to be critical for the growth of melanoma cells [70], and is upregulated in lung cancers [71,72] and colorectal cancer [73]. TIA1 and TIAR are mutated across multiple cancer types and probably function as tumour suppressors [94,145]. Low expression of TIA1 and TIAR correlates with poor prognosis in patients with lung squamous cell carcinoma [75].

With regard to the ZFP36 AU-RBP gene family, perhaps the strongest evidence for a link with tumourigenesis has come from the observation that deletion of both *zfp36pl1* and *zfp36l2* in mice leads to T lymphoblastic leukaemia [82]. Many studies in human cancers have also reported that ZFP36 is underexpressed in different cancers [77,78,79,80]. A general paradigm has emerged consistent with ZFP36 family proteins functioning as tumour suppressor genes. Genome-wide sequencing studies that identified ZFP36L1 as a driver gene in breast cancer [83] and multiple myeloma [84], identifying inactivating mutations in the *ZFP36L1* gene, provided more recent evidence supporting this hypothesis. Based on these observations, a few studies have also reported strategies for inducing ZFP36 family protein expression as novel anti-cancer therapies [146,147]. However, although ZFP36L1 has generally been considered a tumour suppressor gene, in acute myeloid leukaemia cells carrying the AML-ETO1 translocation, ZFP36L1 is overexpressed and is associated with increased proliferation and inhibition of differentiation [148]. More recently, CRISPR Cas9 knockout of ZFP36L1 in chronic myeloid leukaemia cells lines reduced cell growth [85]. These results highlight possible cell context-specific effects of ZFP36L1 on cell growth and proliferation. Like ZFP36L1, ZFP36L2 has also been indicated to play oncogenic and tumour suppressive roles in different cancer types. Specifically, ZFP36L2 was indicated as a tumour suppressor through pan-cancer whole-genome sequencing techniques highlighting ZFP36L2 to be significantly mutated in metastatic tumours [89]. Conversely, ZFP36L2 has been reported to have oncogenic activity in gastric cancer [87] and pancreatic cancer [88], suggesting that ZFP36L2 may adopt cell type-specific functions of which the biological relevance should be further investigated.

## 9. Conclusions and Future Directions

Hitherto, it has been assumed that the important biological role(s) of AU-RBPs (Table 1) are principally related to their well-characterized functions in post-transcriptionally regulating mRNAs by binding to AREs in the 3′UTR of certain mRNAs and mediating either degradation or localization and/or effecting mRNA translation. However, emerging evidence presented here has also highlighted the multi-faceted roles of AU-RBPs (Table 1), particularly those involved in maintaining genome integrity, which may also be important in normal biological processes. This is highlighted by their roles in post-transcriptional regulation of key regulators and mediators of the DDR through canonical mRNA binding of ARE regions in the 3′UTR. Furthermore, non-canonical protein-protein interactions, whereby AU-RBPs are direct targets of DDR components for PTMs, also influence their subsequent activity in response to genotoxicity. Nucleocytoplasmic shuttling capabilities of all the AU-RBPs described in this review is an intriguing area for further study, although, most of the nucleocytoplasmic shuttling of AU-RBPs have been reported to maintain genome integrity through mRNA interactions. A number of AU-RBPs described here can also bind DNA, which presents the prospect that they may also cooperate in DNA repair, as has been discussed for the AU-RBP AUF1. Therefore, it would be of interest to gain further insight into the capabilities of AU-RBPs to localize to sites of DNA damage. There is increasing evidence that dysfunctional AU-RBPs are associated with cancer. Further understanding of the molecular mechanisms and signaling involved with these multi-functional AU-RBPs may elucidate additional insights into their links to genome integrity and may uncover novel therapeutic strategies for targeting these proteins in cancer.

## Figures and Tables

**Table 1:** Multi-faceted roles of AU-rich RNA binding proteins in maintaining genome integrity.

Name (Aliases) AU-RBPs	Chromosome Location	Cell Replication Stress/DNA Damage Role	Role in Cancer—Selected Publications
*AUF1 (HNRNBP, HNRPD)*	4q21.22	Depletion of HNRNPD impairs homologous recombination by inhibiting DNA-end resection and inducing R-loop accumulation [48]	AUF1 is overexpressed in esophageal squamous cell carcinoma (ESCC) patient tissue samples [49] AUF1 overexpression promotes colorectal cancer (CRC) progression [50]
*HuR (ELAVL1)*	19p13.2	HuR reduces radiation-induced DNA Damage by enhancing the expression of ARID1A [51] poly(A)+ RDH mRNAs are post-transcriptionally regulated by HuR in cell stress conditions [52] Component of R loop interactome [53] HuR facilitates cellular senescence through posttranscriptional regulation of TIN2 mRNA [54] HuR stabilizes TFAM mRNA in an ATM/p38-dependent manner in irradiated cells [55] PARG mRNA post-transcriptional regulation by HuR facilitates DNA Repair [56] Chk2 phosphorylates HuR following ionising radiation [57] Double-stranded breaks induce CHK2-mediated phosphorylation of HuR in B lymphocytes [58] HuR functions in oxidative stress pathways [59] HuR post-transcriptionally regulates BRCA1 [60] HuR enhances p53 translation in response to ultraviolet light irradiation [61] HuR regulates p21 mRNA stabilisation by UV light [62] HuR regulates cyclin A and cyclin B1 mRNA stability during cell proliferation [63]	HuR is elevated in clinical ductal invasive carcinoma (DIC) and ductal carcinoma in situ (DCIS) breast cancer samples [64,65] Elevated HuR cytoplasmic expression is a possible indicator of poor prognosis in breast cancer patients that received paclitaxel- and anthracycline-based neoadjuvant chemotherapy (NACT) [66] HuR is an oncogenic driver for malignant peripheral nerve sheath tumours (MPNSTs) [67]
*KHSRP (KSRP)*	19p13.3	DNA damage response phosphorylates KHSRP leading to direct binding to pre-miRNAs promoting miRNA biogenesis [68,69]	KHSRP critical for the growth of melanoma cells [70] KHSRP upregulated in lung cancers [71,72] KHSRP upregulated in colorectal cancer [73]
*TIA1 (TIA-1)* *TIAR (TIAL1)*	2p13.3 10q26.11	TIAR restricts G2/M transition under conditions of replication stress [74]	TIA1 and TIAR function as tumour suppressors and low expression correlates with poor prognosis in patients with lung squamous cell carcinoma [75]
*ZFP36 (TTP, TIS11)*	19q13.2	Modulation of ATR-CHK1 activation upon DNA damage through stabilisation of claspin mRNA [76]	ZFP36 is suppressed in many cancers [77] ZFP36 underexpressed in cancers of the breast, lung and liver [78,79,80]
*ZFP36L1 (TIS11b)*	14q24.1	Early developing T cells in *zfp36pl1 zfp36l2* double knockout showed increased levels of DNA double-stranded breaks marker γH2AX [81]	Deletion of *zfp36pl1* and *zfp36l2* in mice leads to T lymphoblastic leukaemia [82] *ZFP36L1* identified as a breast cancer driver gene [83] *ZFP36L1* identified as a multiple myeloma driver gene [84] CRISPR-KO of *ZFP36L1* reduces cell growth in chronic myeloid leukaemia cells [85]
*ZFP36L2 (TIS11d)*	2p21	ZFP36L2 is essential for cisplatin-induced DNA damage-induced S-phase arrest [86] Early developing T cells in *zfp36pl1 zfp36l2* double knockout showed increased levels of DNA double-stranded breaks marker γH2AX [81]	ZFP36L2 is overexpressed in gastric cancer [87] ZFP36L2 is overexpressed pancreatic cancer [88] ZFP36L2 is a prevalent driver gene in metastatic solid tumours [89]
